# Multi-Level Interactions Between Heat Shock Factors, Heat Shock Proteins, and the Redox System Regulate Acclimation to Heat

**DOI:** 10.3389/fpls.2015.00999

**Published:** 2015-11-17

**Authors:** Nicky Driedonks, Jiemeng Xu, Janny L. Peters, Sunghun Park, Ivo Rieu

**Affiliations:** ^1^Department of Molecular Plant Physiology, Institute for Water and Wetland Research, Radboud University, Nijmegen, Netherlands; ^2^Department of Horticulture, Forestry and Recreation Resources, Kansas State University, Manhattan, KS, USA

**Keywords:** heat response, heat shock factor, heat shock protein, reactive oxygen species, ROS scavenging, signaling, interaction, cross-talk

## Abstract

High temperature has become a global concern because it seriously affects the growth and reproduction of plants. Exposure of plant cells to high temperatures result in cellular damage and can even lead to cell death. Part of the damage can be ascribed to the action of reactive oxygen species (ROS), which accumulate during abiotic stresses such as heat stress. ROS are toxic and can modify other biomacromolecules including membrane lipids, DNA, and proteins. In order to protect the cells, ROS scavenging is essential. In contrast with their inherent harms, ROS also function as signaling molecules, inducing stress tolerance mechanisms. This review examines the evidence for crosstalk between the classical heat stress response, which consists of heat shock factors (HSFs) and heat shock proteins (HSPs), with the ROS network at multiple levels in the heat response process. Heat stimulates HSF activity directly, but also indirectly via ROS. HSFs in turn stimulate the expression of HSP chaperones and also affect ROS scavenger gene expression. In the short term, HSFs repress expression of superoxide dismutase scavenger genes via induction of *miRNA398*, while they also activate scavenger gene expression and stabilize scavenger protein activity via HSP induction. We propose that these contrasting effects allow for the boosting of the heat stress response at the very onset of the stress, while preventing subsequent oxidative damage. The described model on HSFs, HSPs, ROS, and ROS scavenger interactions seems applicable to responses to stresses other than heat and may explain the phenomenon of crossacclimation.

## The Heat Response

Plants are continuously exposed to biotic and abiotic stress factors, such as herbivory, pathogen attack, drought, salinity and extreme temperatures. These challenges pose a serious threat to their growth and reproduction and as such affect agricultural yields. With considerable advances in pest and disease management, abiotic factors are now thought to be the primary cause for crop losses worldwide (Wang et al., 2003; Suzuki et al., 2014). In case plants cannot prevent an abiotic stress factor from affecting organismal homeostasis (i.e., escape or avoid internal stress), they may adapt their metabolism to acquire a certain level of tolerance (Larkindale and Knight, 2002; Valliyodan and Nguyen, 2006; Munns and Tester, 2008; Krasensky and Jonak, 2012).

Heat stress can be defined as a rise in temperature beyond a threshold level for a period of time, sufficient to cause irreversible damage to plant growth and development (Wahid et al., 2007). Sudden rises in temperature to high levels may lead to cell death within a few minutes as a consequence of extensive protein denaturation and aggregation and loss of membrane integrity (Schöffl et al., 1999; Wahid et al., 2007). Furthermore, prolonged exposure to moderately high temperatures can lead to reduced cellular function and overall plant fitness (Bokszczanin et al., 2013). An important process in this respect is the accumulation of reactive oxygen species (ROS), formed as a by-product in various aerobic metabolic pathways in different cellular compartments such as chloroplasts, mitochondria and peroxisomes (del Rio et al., 2006; Navrot et al., 2007) and probably also in the apoplast through the activation of NADPH oxidases (Gechev and Hille, 2005; Torres and Dangl, 2005; Miller et al., 2009; Wang et al., 2014a). Under steady state conditions, ROS molecules are formed as quickly as they are scavenged by anti-oxidative defense mechanisms, but this equilibrium is perturbed by abiotic stress factors such as heat (Foyer and Noctor, 2005). There is ample evidence that, when plants are exposed to heat, ROS production rapidly becomes excessive (Morgan et al., 1986; Dat et al., 1998; Vacca et al., 2004; Volkov et al., 2006; Bhattacharjee, 2012, 2013; Chou et al., 2012; Hasanuzzaman et al., 2012, 2013; Wu et al., 2012; Hossain et al., 2013; Das and Roychoudhury, 2014; Mostofa et al., 2014). This causes cellular damage to membranes, proteins, lipids, organelles, and DNA (Baker and Orlandi, 1995; O’Kane et al., 1996; Giardi et al., 1997; Larkindale and Knight, 2002; Volkov et al., 2006; Wu et al., 2012; Bokszczanin et al., 2013). In order to prevent cell damage and regain redox homeostasis, one of the responses to heat is the hyper-activation of the ROS scavenging machinery. The expression and protein level of genes responsible for ROS scavenging are induced under heat stress in many different plant species (Chao et al., 2009; Chou et al., 2012; Mittal et al., 2012; Suzuki et al., 2013) and has been associated to basal heat tolerance (Rui et al., 1990; Badiani et al., 1993; Gupta et al., 1993; Sairam et al., 2000; Almeselmani et al., 2006; Kang et al., 2009; Bhattacharjee, 2012; Wang et al., 2014c). Furthermore, the induction of scavenging genes was significantly stronger in heat tolerant genotypes than that of sensitive ones (Rainwater et al., 1996), and improvement of plant heat stress tolerance has been achieved by increasing antioxidant enzymes activities (Rui et al., 1990; Badiani et al., 1993; Gupta et al., 1993; Sairam et al., 2000; Almeselmani et al., 2006; Wu et al., 2012; Chen et al., 2013). Taken together, this shows the importance of ROS scavenging in the heat-stress response.

In contrast to their harmful character, however, ROS are also considered as important signal molecules. Cells are capable of rapid and dynamic production and control of several forms of ROS, enabling a tight local control in the cell as well as more holistic control of the entire plant (Vranová et al., 2002; Mittler et al., 2011; Petrov and Van Breusegem, 2012). Therefore, they are thought to be involved in the transduction of intracellular and intercellular signals controlling gene expression and activity of anti-stress systems (Desikan et al., 2001, 2004; Apel and Hirt, 2004; Foyer and Noctor, 2005; Torres and Dangl, 2005; Miller et al., 2009; Galvez-Valdivieso and Mullineaux, 2010; Mittler et al., 2011; Kreslavski et al., 2012). Indeed, NADPH oxidase activity is rapidly induced upon heat (Miller et al., 2009) and the mutation of *RBOHB* makes *Arabidopsis* seedlings more sensitive to heat (Larkindale et al., 2005; Wang et al., 2014a).

One of the best studied anti-stress mechanisms is the production of heat shock proteins (HSPs) upon exposure to high temperatures (Wang et al., 2004). By acting as molecular chaperones, HSPs prevent deleterious protein conformations and eliminate non-native aggregations, which are formed during stress (Vierling, 1991; Boston et al., 1996; Morimoto, 1998). The expression of HSPs and other heat-responsive genes is regulated by heat shock factors (HSFs; Kotak et al., 2007) through their association to a palindromic binding motif (5′-nAGAAnnTTCTn-3′) in the promoter region of the heat-responsive genes: the heat shock element (HSE; Pelham, 1982; Scharf et al., 2012). Activation of HSFs upon stress occurs via a multistep process involving homotrimer formation and acquisition of transcriptional competence for target gene induction (Liu et al., 2013).

Clearly, both the activation and production of HSFs/HSPs and the increase in ROS/scavenging activity belong to the major responses of plants to heat stress and play important roles in acclimation. A number of recent genetic and biochemical studies, however, indicate that there are complex interactions between these responses. This review describes the evidence for crosstalk between HSFs, HSPs, ROS, and ROS scavenging enzymes at various points in the heat stress response pathway and presents a model with a timing component.

## Activation of HSFs by ROS

In non-stressed situations, the HSFs are located in the cytoplasm for most eukaryotes, in an inactive monomeric form due to association with HSP70, HSP90, and potentially other proteins (Morimoto, 1998; Schöffl et al., 1998). According to the chaperone titration model, heat results in a higher load of denatured proteins, which pulls HSPs away from HSF complexes through competitions to act as molecular chaperones. This then leads to the release of HSFs, which form trimers and relocate to the nucleus to activate expression of *HSP* and other heat-responsive genes (Zou et al., 1998; Volkov et al., 2006).

A number of studies, however, report that expression of heat-responsive genes is also increased upon application of the ROS H_2_O_2_ (Uchida et al., 2002; Wahid et al., 2007; Banti et al., 2008). For example, *AtHSP17.6* and *AtHSP18.6* achieved similar expression levels through heat treatment as they do through H_2_O_2_ application at room temperature (Volkov et al., 2006). Several hypotheses have been formulated that suggest that heat can indirectly activate HSFs via the action of ROS.

Firstly, damaging amounts of heat-induced ROS also induce protein denaturation. In this way ROS enhances dissociation of the HSP–HSF complex, as described by the titration model (Schöffl et al., 1998). Secondly, and similar to what was found for mammalian and *Drosophila* HSFs, it has been proposed that certain plant HSFs act as H_2_O_2_ sensors (Ahn and Thiele, 2003; Miller and Mittler, 2006). Among all the ROS molecules, H_2_O_2_ plays a key role in signaling due to its moderate reactivity and thus relatively long lifetime (Vranová et al., 2002). In addition, H_2_O_2_ can bypass membranes easily, making it a good candidate to function as a signaling molecule (Petrov and Van Breusegem, 2012). Miller and Mittler (2006) suggested that H_2_O_2_ might directly modify HSFs and induce HSF trimerization. Indeed, both heat and oxidative stresses result in the formation of high molecular weight HSE-binding complexes and the formation of these complexes has been shown to be a signature of early HSFA1a/A1b-dependent gene expression in heat-stressed leaves of *Arabidopsis* (Lohmann et al., 2004; Volkov et al., 2006). *In vitro* and *in vivo* studies confirmed activation of AtHSFA1a via trimerization in response to heat and H_2_O_2_ stress but also via pH alterations (Liu et al., 2013). HSFA1a, purified from *E. coli*, sensed the different stresses directly in a redox dependent fashion. *In vitro* stress treatments caused monomer-to-trimer transitions of HSFA1a, while the presence of the reducing agent dithiothreitol reversed this action. Although the study suggested a redox dependent fashion for HSF trimerization for all three stresses, the exact mechanism of action is still unclear. There is empirical evidence that the transcription factors may be sensitive to H_2_O_2_ via “single-Cys” or “two-Cys” redox sensory mechanisms (Mittler et al., 2011). These cysteine residues are typically responsive to oxidative stress. HSFA1a contains one Cys residue located at the *N*-terminal portion of the trimerization domain (Hübel and Schöffl, 1994). *N*-terminal deletions of HSFA1a negatively affected the sensing of H_2_O_2_ and pH changes, which suggests that trimerizations were induced by HSF conformational changes (Liu et al., 2013). In addition, Giesguth et al. (2015) recently showed that an HSFA8 Cys residue is responsible for translocation to the nucleus upon oxidative stress: H_2_O_2_ treated protoplasts showed cytosol-to-nucleus translocations of the wild-type HSFA8, but not of the HSFA8C24S mutant variant (Giesguth et al., 2015). Interestingly, however, the *N*-terminal deletion of HSFA1a did not inhibit heat sensing. This shows that activation of this particular transcription factor is stress-specifically regulated despite a common dependency on oxidative activity (Mittler et al., 2011). Notably, all stress treatments induced of HSFA1a binding to the *HSP18.2* and *HSP70* promoter, as detected by both formaldehyde cross-linking and chromatin immunoprecipitation, which paralleled the mRNA expression of these HSFA1a target genes (Volkov et al., 2006; Liu et al., 2013).

In addition to the above two processes, cellular communication between ROS and HSFs may involve mitogen-activated protein kinases (MAPK). HSF phosphorylation has been observed in yeasts and mammals (Chu et al., 1996; Knauf et al., 1996; Kim et al., 1999) and might thus occur in plants as well (Link et al., 2002). Indeed, *Arabidopsis* HSFA2 was found to be phosphorylated by MPK6 on T249 after heat treatment, and this was associated with subsequent intracellular localization changes (Evrard et al., 2013). Furthermore, MPK3- and MPK6-dependent phosphorylation of AtHSFA4A Ser309 and physical interaction between the proteins was reported recently (Pérez-Salamó et al., 2014). Activated HSFA4A in turn controlled the transcription of *HSP17.6A* (Pérez-Salamó et al., 2014). In tomato, heat-induced MAPKs were shown to transduce heat stress signals via HSFA3 (Link et al., 2002). In Arabidopsis, the same MAPKs that phosphorylate HSFs, namely MAPK3 and MAPK6, have been shown to be activated by H_2_O_2_ (Kovtun et al., 2000; Moon et al., 2003; Rentel et al., 2004). However, despite the presence of putative phosphorylation sites in tomato HSFA1, no heat-induced phosphorylation of this HSF was observed. Also, the phosphorylation site in AtHSFA4 was not conserved in HSFA4A proteins of citrus, grapevine and poplar (Pérez-Salamó et al., 2014). Taken together, this implies that both HSF oxidation and ROS-dependent phosphorylation can play a role in HSF activation, but that the latter is not a general signaling mechanism.

## HSF–ROS Scavenging Gene Interactions

In addition to activation of HSFs by ROS signaling, evidence for interaction between HSFs and ROS scavenging genes has also been obtained. The expression of *APX1* was found to be regulated by *HSFA2*: overexpression of *HSFA2* resulted in increased expression of *APX1*, while *AthsfA2* knock out mutants showed a reduced expression of *APX1* (Li et al., 2005). In agreement with this, AtHSFA2 overexpression lines showed increased heat and oxidative stress tolerance (Li et al., 2005). Expression of a dominant-negative construct for *AtHSFA4a* prevented the accumulation of *APX1* transcripts (Pnueli et al., 2003; Apel and Hirt, 2004; Mittler et al., 2004; Davletova et al., 2005). Interestingly, the *AtHSFA4a* dominant-negative construct also prevented accumulation of the H_2_O_2_-responsive zinc-finger protein ZAT12, which is required for *APX1* expression during oxidative stress. The *ZAT12* promoter contains HSE binding sites (Rizhsky et al., 2004) and therefore, HSFA4a might directly interact with the *ZAT12* promoter (Davletova et al., 2005). However, HSEs are also present in the promoter region of the *APX1* gene itself, suggesting that direct activation via HSFs is also possible (Storozhenko et al., 1998; Panchuk et al., 2002). Using *Pennisetum glaucum APX1* and a *PgHSFA*, a specific binding interaction between the *APX1* HSE and HSF was confirmed, via *in vitro* gel shift assays as well as their expression patterns over time (Reddy et al., 2009).

Although *APX1* has been shown to be a central component of the *Arabidopsis* ROS network (Davletova et al., 2005), *APX2*, another isoform also localized in the cytosol, revealed a stronger induction by heat stress (Panchuk et al., 2002). AtHSFA2 has also been found to act as an *APX2* activator (Schramm et al., 2006; Nishizawa et al., 2008). Transcription level comparison between wild-type and *athsfa2* knock out plants revealed that transcripts of *APX2* were absent in heat shock induced leaves of the knock out background, but present in the wild-type plants (Schramm et al., 2006). Deletion analyses of the promoter region of *APX2* functionally mapped the HSFA2 binding sites to HSEs near the transcription start site (Schramm et al., 2006).

In addition, Nishizawa et al. (2006) and Banti et al. (2010) found strongly enhanced expression of galactinol synthase (*GolS1* and *GolS2*) ROS scavenging genes in an *HSFA2* overexpressing line.

Combining these results, HSFA2 seems to play a central role in ROS scavenger expression and thus constitute an important link between heat shock and oxidative stress responses.

## HSP Chaperones Support ROS Scavenging Activity

Heat shock proteins function as molecular chaperones and play an important role in stress tolerance. In tomato, overexpression of the *LeCDJ1* DnaJ protein coding gene (also known as J-protein or HSP40; Qiu et al., 2006) resulted in improved thermotolerance, accompanied by increased APX and superoxide dismutase (SOD) activity after heat stress and reduced accumulation of O_2_^–^ and H_2_O_2_. Despite the higher APX and SOD activity, transcription of the corresponding genes was not enhanced in the transgenic plants. Therefore, the influence of DnaJ proteins on APX and SOD activity was proposed to be post-transcriptional, due to their functionality as chaperones. Other studies have found similar effects of HSPs on ROS scavenging proteins upon heat stress. In *Arabidopsis*, overexpression of *RcHSP17.8* enhanced SOD activity (Jiang et al., 2009) whereas overexpression of *ZmHSP16.9* in tobacco enhanced POD, CAT, and SOD activity (Sun et al., 2012). Altogether, it may be hypothesized that the HSP proteins positively affect thermotolerance by protecting ROS scavenging protein conformation and activity, resulting in a lower ROS concentration (Kong et al., 2014a).

An alternative link between DnaJ proteins and ROS scavenging was suggested by Zhou et al. (2012). They showed that *Arabidopsis* AtDjB1 knockout plants (*atj1-1*) were more sensitive to heat stress than wild-type plants. After heat shock, the knockout plants showed an increased concentration of H_2_O_2_ and other oxidative products as well as a decreased concentration of the antioxidant ascorbate (ASC; Mittler et al., 2004; Zhou et al., 2012). The viability of *atj1-1* knockout seedlings after heat stress was rescued by exogenous ASC application. This suggests that lower concentrations of the antioxidant in *atj1-1* knockout plants resulted in increased H_2_O_2_ concentrations leading to a decreased thermotolerance (Zhou et al., 2012). As the underlying cause, the authors hypothesize a link with the electron transport chain (ETC). AtDjB1 directly interacts with a mitochondrial HSP70 and stimulates ATPase activity (Zhou et al., 2012), a mechanism which is conserved among several kingdoms (Qiu et al., 2006). AtDjB1 knockout potentially leads to the accumulation of cellular ATP, which feedback inhibits ETC. Because the last step of ASC synthesis is linked to the ETC (Bartoli et al., 2000), decreased ETC results in decreased ASC concentration and, consequently, the accumulation of H_2_O_2_ (Zhou et al., 2012).

Although it is unclear whether there is a specific interaction between HSPs and the ROS scavenging machinery or that HSPs generally maintain protein functions, these results indicate that upon heat stress, accumulation of ROS is reduced via HSP supported ROS scavenger activity.

## A Positive Feedback Loop Including HSFs, ROS Scavenging Genes, and miRNA398

In contrast to the positive effects of ROS reducing mechanisms on heat stress tolerance, an *Arabidopsis* study provided evidence linking enhanced ROS accumulation to higher stress tolerance (Guan et al., 2013). The research indicated the existence of a positive a feedback loop, whereby heat and ROS allow for further ROS accumulation, depending on the actions of microRNA398 (*miRNA398*). *miRNA398* expression was found to be induced within 1 h and reach its peak 2 h after heat stress. The *miRNA398* promoter region contains a putative HSE, and chromatin immune-precipitation assays revealed direct binding of HSFA1b and HSFA7b to the HSE promoter region under heat stress. Thus, association of these HSFs to the promoter region seems to be responsible for the induction of this miRNA upon heat stress (Guan et al., 2013). miRNA398 negatively regulates the expression of three target genes: *CSD1*, *CSD2*, and *CCS* (Guan et al., 2013). *CSD1* and *CSD2* genes are isoforms of copper/zinc-SOD scavenging genes which are located in the cytoplasm and chloroplasts, respectively (Bowler et al., 1992; Kliebenstein et al., 1998) and *CCS* is a copper chaperone encoding gene, which delivers copper to both *CSD* genes (Cohu et al., 2009). Consequently, *CSD1*, *CSD2*, and *CCS* are down-regulated during heat stress, allowing further ROS accumulation. This pathway acts in an autocatalytic manner, as H_2_O_2_ in turn promotes expression of various HSFs, including *HSFA7b* (Guan et al., 2013). Accumulation of ROS seems to be an unfavorable response for the plant to survive heat stress. However, comparison of wild-type and *csd1*, *csd2*, and *ccs* mutants plants revealed higher heat tolerance in mutant plants while transgenic plants over-expressing *miR398*-resistant versions of *CSD1*, *CSD2*, or *CCS* were hypersensitive to heat stress (Guan et al., 2013).

These unexpected outcomes may be explained by the increases in oxidative power for helping activate the primary set of HSFs at the start of the heat response. In contrast to Guan et al. (2013); Sunkar et al. (2006) found that lack of *miRNA398* enhances tolerance to some other stress factors, high light and chemically induced ROS, via enhanced expression of *CSD1* and *CSD2* (Sunkar et al., 2006). Therefore, it seems that the benefit of reduced SOD activity is heat-specific, potentially due to the importance of high HSF activity in the first hours of the response to this stress.

## A Multi-Level Interaction Model

**FIGURE 1:**
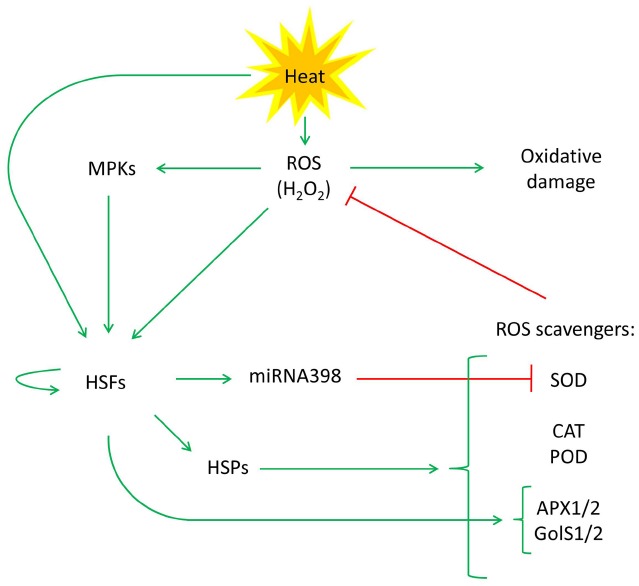
**Model describing crosstalk between the HSF/HSP and ROS pathways in the heat stress response.** In addition to directly activating HSFs, high temperature leads to the accumulation of ROS. This, in turn, leads to a further activation of HSFs, either directly or indirectly via activation of a MAPK pathway. The HSFs bind to HSE in the promoter region of *HSF*, *HSP*, *miRNA398*, and ROS scavenging genes. The miRNA398-dependent down-regulation of a subset of SOD scavengers might play a role in the rapid ROS accumulation upon exposure to heat. This would support the activation of HSFs and thereby boost the induction of the heat-stress response in the short term. In the longer term, the induction and stabilization of other scavengers would start to suppress ROS levels to avoid excessive cellular damage.

A number of recent studies have provided evidence for connections between HSFs, HSPs, ROS, and ROS scavengers upon heat stress. Here, we propose a comprehensive model on the relations between the various components to explain a large proportion of the observations (Figure 1). Through contrasting effects on ROS scavenging activity, heat shock induces a short-term positive (roughly, within the first few hours of heat stress) and a long-term negative feedback loop (after the first few hours of heat stress) on the HSF signaling pathway. The proposed complexity of the heat-stress response network is mirrored in some of the counter-intuitive observations, such as enhanced heat tolerance in certain scavenger mutants (Rizhsky et al., 2002; Vanderauwera et al., 2011). However, analogous to the proposed *miRNA398* mechanism, constitutively, slightly elevated ROS levels in such mutants may result in a primed state and, as a consequence, a stronger and/or faster response to a heat treatment. Guan et al. (2013) indeed showed that knockout mutations in *CSD1* and *CSD2* were accompanied by constitutively higher levels of HSF and HSP transcripts. In accordance, the importance of ROS at early heat response was shown by Volkov et al. (2006): a rapid oxidative burst of ROS during the first 15 min of the heat shock stimulates HSF DNA-binding and is essential for the induction of heat responsive gene expression, e.g., of *HSPs* and *APX2* (Volkov et al., 2006). The typical “late” high mobility HSE-binding complexes, formed after 2 h, were shown to be ROS independent (Lohmann et al., 2004; Volkov et al., 2006), which is in accordance with the production of anti-oxidants and ROS scavengers reducing the ROS overload after the early onset of the HSR (Chaitanya et al., 2002; Wahid et al., 2007; Frank et al., 2009; Dong et al., 2015). Nevertheless, if plants are continuously exposed to heat stress, the activity of some antioxidants and scavengers, e.g., APX and CAT, decreased after 3 days of heat stress in tomato, alfalfa and tobacco cell cultures (Wu et al., 2012; Li et al., 2013; Sgobba et al., 2015). The changes of these components of the antioxidant system were ascribed to the impaired health and growth of plants under long term heat stress and are different from short term heat stress (de Pinto et al., 2015).

Importantly, the model described here refers specifically to the complexity of events after a short term heat shock; its applicability to other types of heat stress, e.g., mild levels of heat stress, which only affect plant physiology in the long term, is not evident and more research will be necessary in order to clarify how the HSF/HSP and ROS systems behave under those circumstances. Also, it should be noted that the proposed model is not stand-alone and will interact with other factors, such as phytohormones. Abscisic acid (ABA), salicylic acid (SA) and ethylene have all been implicated in the heat response and can induce the production of ROS (Kwak et al., 2006; Foyer and Noctor, 2009). While a number of phytohormone-related mutants show impaired tolerance to heat (Larkindale et al., 2005), application of these hormones may enhance thermotolerance via an effect of ROS. SA application, for example, enhanced SOD activity and *HSP* expression during heat stress (Clarke et al., 2004; He et al., 2005). Dedicated analysis of the role of hormones during the first hours of heat treatment should clarify their putative positions in the response model.

The model may well have broader applicability then to the heat response only (Jiang and Zhang, 2002; Jammes et al., 2009; Bartoli et al., 2013; Wang et al., 2014b; Hossain et al., 2015). Not only are ROS accumulation, signaling and scavenging thought to occur and play a role in myriad other stress responses (Mittler, 2002; Hossain et al., 2015), but so is HSP activity (Pastori and Foyer, 2002; Banti et al., 2008; Pucciariello et al., 2012). HSPs are also induced upon water stress, salinity and osmotic stress, cold, anoxia, UV-B light, and oxidative stress (Vierling, 1991; Waters et al., 1996; Wang et al., 2004; Loreti et al., 2005; Swindell et al., 2007). Furthermore, overexpression of various HSFs enhanced tolerance to abiotic stresses other than heat, including salt, drought, osmotic, and anoxic stress (Bechtold et al., 2013; Chauhan et al., 2013; Shen et al., 2013; Pérez-Salamó et al., 2014). Also, tomato plants overexpressing the DnaJ/HSP40 *LeCDJ1* showed both higher heat and chilling tolerance (Kong et al., 2014a,b) and overexpression of BRZ-INSENSITIVE-LONG HYPOCOTYLS 2 (BIL2), a mitochondrial-localized DnaJ/HSP40 family member, enhanced resistance against salinity and high light stress (Bekh-Ochir et al., 2013). The role of both the oxidative stress and HSF/HSP systems in multiple stress responses might explain the phenomenon of cross-acclimation, where exposure to a certain stress factor improves tolerance to a subsequent different stress factor (Banti et al., 2008, 2010; Chou et al., 2012; Byth-Illing and Bornman, 2013; Hossain et al., 2015).

### Conflict of Interest Statement

The authors declare that the research was conducted in the absence of any commercial or financial relationships that could be construed as a potential conflict of interest.
